# The Case for the Members

**Published:** 1909-11-27

**Authors:** 


### The Case for the Members.

At the annual general meeting of the Royal College of Surgeons the usual motion—one which bids fair to become a hardy annual—relative to the status of members was moved, seconded, and passed without a dissenting vote, though it would be a mistake to say it was passed unanimously, since some at least of those present abstained from recording their vote. No one knowing the condition of affairs can doubt that members of the College have a legitimate grievance, in so far as they have no voice in the politics of the College, or in the administration of its affairs. Such privileges of collegiate citizenship are reserved for Fellows who number less than one-seventeenth of the professional community which the Council is supposed to represent. That being the case, it is only natural that Members should feel aggrieved at being treated virtually as outsiders. Their case has been put forcibly at many annual general meetings, and there can be little doubt that the majority of Fellows sympathise with them and wish to see these grievances removed. But between wishing and realising lies a wide gulf, and the Council holds out no hopes that it is likely to be bridged within the near future.

We do not consider that any practical purpose is served by constantly bringing forward this motion which in its very essence is an indictment of the capacity of the Council to represent the College. If the members as a body feel their position acutely (and we think they do) it is desirable that they should attend the usual meeting in force, and by the weight of their numbers dignify this solemn annual protest. Last week's meeting was attended by a small body of men whose claim to voice the opinions and wishes of the vast mass of their colleagues in general practice rests on as flimsy a basis as the claim of the Council to represent the College. One of the speakers pithily remarked that since he gained his diploma the College has done nothing for him, and the majority of Members present seemed to be in hearty agreement to that expression of opinion. In a measure that statement epitomises the situation. The College does nothing for the general practitioner—the general practitioner nothing for the College. Both statements are true in one sense, and in another they are absolutely false, for the general practitioner, at least so far as the public is concerned, has the weight of the College behind him just as the Council has behind it (whether in reality or shadow does not, for the present argument, matter very much) the solid mass of medical men in this country. We have no doubt that the influence and the good works of the Council would be enormously increased if the Members were represented upon the Administration Board; but such representation, in the nature of things as they are, must be by act of courtesy, not as a matter of right. Nor is it to be expected that the Council, even when its strength has been augmented by the accession of representatives of the Members, should act as a committee of an associational branch, and deal with matters which do not directly affect the College. The speeches in favour of the protesting motion and in support of the other two motions brought forward at the general meeting, seem to us to be in the nature of special pleadings of matters which, however important they may be deemed by members of the profession who have at heart associational ideals, are scarcely such as the body corporate of the College can justifiably deal with or upon which it can express an opinion. It is desirable and necessary that our examining bodies—and the College is principally an examining body—should remain severely outside professional political discussions, especially when they are of a nature that admits of more than one legitimate opinion. When all, however, is said and done the root argument of the case for the Members remains unshaken. Members of the College have rights in virtue of the fact that their numbers invest the College with power and influence; they ought to be represented on the Council and have, at least, some share in the management. It is to be hoped that the Council will secure means to amend the restricting acts which debar it from extending to the majority of English medical men a courtesy which is politically expedient and morally just.

